# Covered or Not, Here I Come: Stanford Type B Aortic Dissection Repair With a Covered and Uncovered Stent Hybrid Technique

**DOI:** 10.7759/cureus.11729

**Published:** 2020-11-27

**Authors:** Taylor S Harmon, Alexander Ghannam, Travis E Meyer, Carissa Concepcion, John Pirris, Jerry Matteo

**Affiliations:** 1 Radiology, University of Florida College of Medicine, Jacksonville, USA; 2 Cardiothoracic Surgery, University of Florida College of Medicine, Jacksonville, USA; 3 Surgery, University of Florida College of Medicine, Jacksonville, USA

**Keywords:** tevar, abdominal aorta, thoracic aorta, bare metal stent, type b aortic dissection, interventional radiology, type a aortic dissection, covered stent, three dimensional printing, aortic dissection

## Abstract

The complications resulting from aortic dissections are often devastating. Historically, when a Stanford B aortic dissection extended into the visceral abdominal aorta, only surgical management was considered to limit visceral organ malperfusion. Complications of surgical management for Stanford B aortic dissections are as high as 50%. The inherently high complication and mortality rate for any acute aortic dissection, in addition to the complication rates resulting from surgical management, have demonstrated poor outcomes. This is especially true when aortic dissections involve the visceral segment, where thoracic endovascular aortic repair (TEVAR) becomes limited or contraindicated. In the last two decades, various approaches for TEVAR have improved in both endograft design and interventional technique. The current literature demonstrates improved outcomes for patients that receive TEVAR for Stanford B aortic dissections, including those that involve the visceral segment. Despite favorable prognostic advancement in TEVAR, the proven management complexity of Stanford B aortic dissections continue to reflect the pitfalls of the endovascular devices that are currently available. We describe a covered and uncovered stent hybrid technique in patients with complicated Stanford B aortic dissections involving the visceral segment, considering these deficiencies. Hundred percent technical success was demonstrated in the short and mid-term surveillance periods.

## Introduction

Aortic dissection is a highly lethal entity that prompts aggressive interventional and surgical management. The age-dependent incidence of aortic dissection is between 3-6/100,000 and 10/100,000 person-years in the elderly [1]. Treatment algorithms have been designed to constitute treatment options based on the type of dissection (Stanford A or B) and the acuity. Though all aortic dissections have a high mortality rate, they can be categorized by sensitivity. Acute Stanford A aortic dissections are the most urgent compared to the less urgent chronic Stanford B aortic dissections. Despite improvements in interventional, surgical, and medical management, morbidity and mortality rates are high. Acute aortic dissections are defined by sudden onset of fewer than 14 days, and chronic dissections are present in patients over a two week time period [2]. Emergent surgical management is reserved for acute Stanford A dissections; as Anagnostopoulos et al. notably reported in 1972, the mortality for acute Stanford A aortic dissections increases 1% per hour without surgical intervention [3].

Acute Stanford A and B aortic dissections have a poor prognosis if not appropriately treated versus chronic aortic dissections [4]. Approximately 20% of patients with acute Stanford type A aortic dissections die en route to the hospital. Moore et al. and Lauterbach et al. described that mortality for any untreated aortic dissection is approximately 25% within six hours and 50% within 24 hours. Within one week, two-thirds of the patients with acute aortic dissections die if untreated. Combined, 75% of patients with acute aortic dissections die within the first two weeks [5,6]. Therefore, a comprehensive and collaborative multispecialty center facilitates the optimally required care necessary to usher the desired prognostic results in patients with acute aortic dissections. 

The International Registry of Aortic Dissection (IRAD) is a consistently referenced registry of 21 centers consecutively enrolling patients with any aortic dissection [7]. According to the IRAD, more than one-fourth of admitted patients with aortic dissections will die during hospitalization [7]. Morbidity and mortality differ between Stanford A and B dissections, where prognosis depends on pre-existing comorbidities and treatment options.

The mortality rate of patients who are limited to treatment with medical management for Stanford A aortic dissections is approximately 60%, versus 25% for surgically managed cases [8]. These patients die of aortic valve dysfunction, visceral malperfusion, pericardial tamponade, myocardial infarction, and aortic rupture. Though less severe in prognosis, the mortality for Stanford B dissections is between 10% and 15% for patients who are medically managed. However, surgical or endovascular therapy is indicated for complications, including progressive abdominal pain, dissection expansion, visceral organ malperfusion, and rarely aortic rupture. The presence of these complications increases the mortality rate for Stanford B aortic dissections by 30% [9].

Though there is a high risk for mortality with Stanford A and B aortic dissections, regardless of acuity or chronicity, surgical management is not always indicated in Stanford B dissections, unlike Stanford A dissections. Therefore, more endovascular treatment options are available for the management of Stanford B aortic dissections. Risk stratifying patients with Stanford B aortic dissections can be beneficial when deciding to employ endovascular management in addition to medical management. Multispecialty care should be utilized for the patient’s benefit, considering the patient’s aortic anatomy, extent of the aortic dissection, patient demographic, symptomatology, and patient comorbidities. 

The demographic of patients who are at high risk for developing a Stanford B aortic dissection are those with hypertension, increased age, and male gender [10]. The ratio of male to female dominance for the development of Stanford B aortic dissections is five to one [11]. Patients who develop Stanford B aortic dissections are typically ten years older than patients with Type A aortic dissections. The peak incidence for the development of Stanford B aortic dissections is between 60-70 years and 50-60 years for the development of Stanford A aortic dissections [11]. Approximately 75% of patients with Stanford B aortic dissections have hypertension [4]. Therefore, adjunct medical management is integral in decreasing the endovascular complication rate of Stanford B aortic dissections. Though age and gender are invariable to the patient, reducing the detriment of hypertension can lead to improved outcomes for endovascular intervention in the extended surveillance period [12].

The management of Stanford B aortic dissections has evolved with the emergence of improved endovascular devices. The development of endovascular aortic repair has progressed to the currently approved Food and Drug Administration (FDA) endovascular devices we have today. Risk stratification should always be considered when repairing Stanford B aortic dissections, most considerably when the visceral aortic segment is involved.

The devices currently approved for thoracic endovascular aortic repair (TEVAR) have favorable outcomes, including lower morbidity, mortality, and patency when compared to surgical intervention [13,14]. On-label devices used to treat Stanford B aortic dissections preserve proximal true lumen patency. However, the distal patency of Stanford B aortic dissections has proven to be challenging. Though the false lumen of most proximal aortic dissections thromboses after endovascular repair, it is typical for dissection expansion into the aortic visceral segment. As a result, the on-label thoracic aortic endograft inherent radial force inadequately retains true lumen patency in the visceral segment. When a repaired or native Stanford B aortic dissection expands into the visceral segment, the options for the restoration of perfusion may be limited to surgical management. This option demands appropriate risk stratification, operational precision, and a thorough understanding of the patient's anatomy when planning a TEVAR. Furthermore, the interventionist must understand the device's limitations when choosing an endovascular device. This will ensure patency and limit the risk of future surgical management.

The treatment of Stanford B aortic dissections is multifactorial. Extended-term patency in the post-procedural setting has proven difficult due to well-documented complications and high patient mortality, despite treatment. However, though the development of endograft technology has decreased patient mortality in the last two decades to treat Stanford B aortic dissections, documented device limitations and complications only contribute to treatment complexity. With the many endovascular options on the market at the interventionist's disposal, careful attention to the appropriate use of these devices can lead to improved patient outcomes. One example may be operator dependent piecemeal placement of endovascular devices, utilized as a reliable method for avoiding infolding and endoleaks when reconstructing aortic bifurcations [15,16]. 

Innovative techniques showing promising technical success have been described to treat Stanford B aortic dissections that extend into the visceral segment. Nienaber et al. first described the Provisional Extension to Induce Complete Attachment (PETTICOAT) technique. The PETTICOAT technique uses an off-label bare-metal stent to exclude the aortic dissected false lumen. By doing so, there is an expansion within the aortic true lumen [17]. The technique was designed to combat an expanding false dissection lumen while preserving true lumen patency [17]. At one year follow-up, there was a 100% true lumen patency of the aortic visceral segment [17]. Our institution uses a combination of on-label devices with a covered and uncovered/open cell stent hybrid technique to achieve true lumen patency. We retrospectively analyzed nine patients from our institution that were treated for complicated Stanford B aortic dissections that extended into the visceral segment.

## Materials and methods

**Figure 1:**
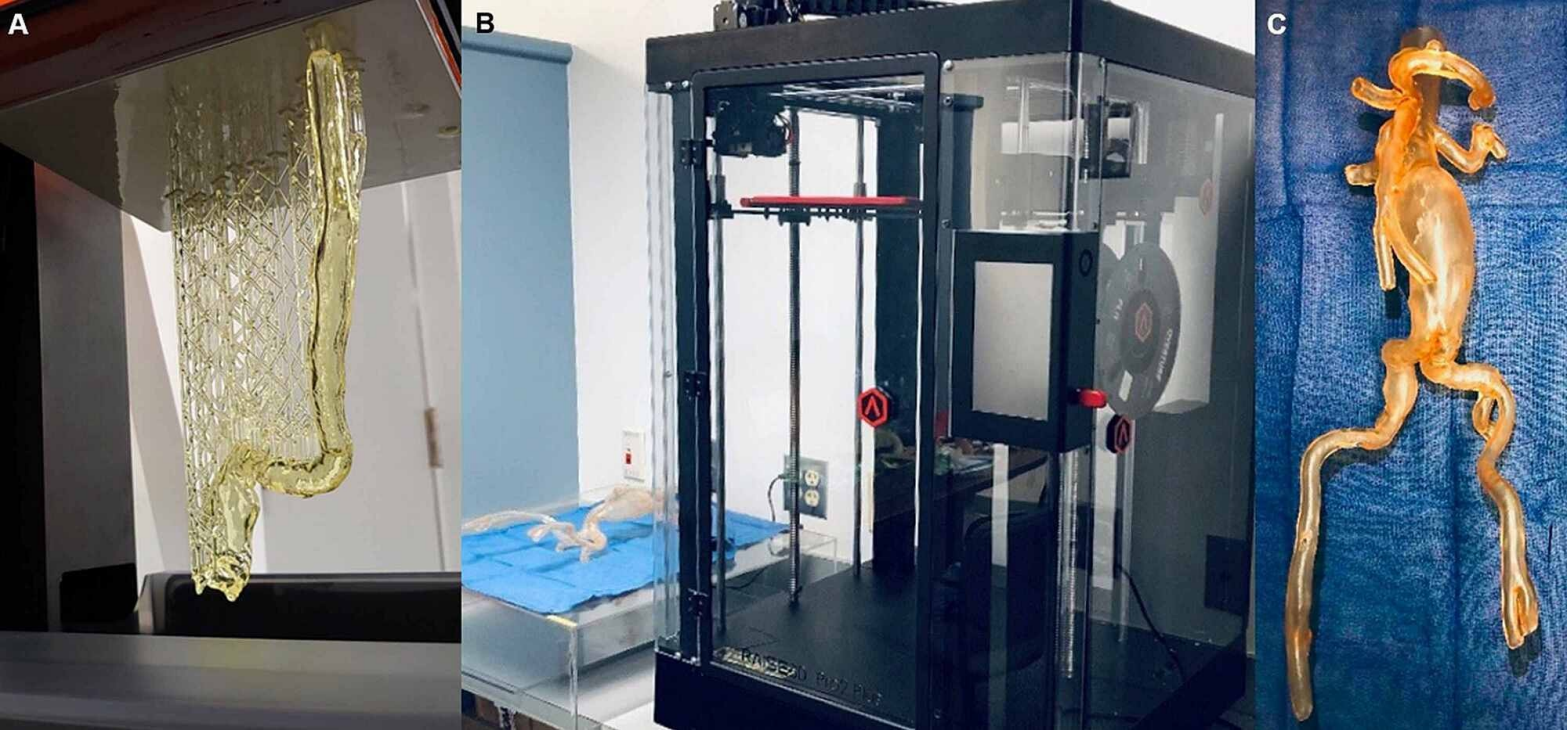
Pre-Procedural Planning With Three-Dimensional Model Rendering The pre-procedural planning of a patient with an aortic aneurysm includes the three-dimensional rendering of the aorta. The patient’s aorta is printed using three-dimension volume software, uploaded from our institutional picture archiving and communication system (A). The printed aorta was cured, shown on a blue towel, next to one of our institution's three-dimensional printers (B). The completely cured three dimensionally printed aorta is shown (C). The printed aortic model shows the aortic aneurysm, visceral segment, and bilateral common iliac arteries. This model will be used to ease in-vitro visualization and simulation before endovascular repair in-vivo.

The treatment success of complex Stanford B aortic dissections is a complicated endeavor that requires careful consideration. Achieving positive outcomes for TEVAR may involve discussing patients in case conferences, an extensive review of pertinent imaging, and at our institution, the utilization of three-dimensional anatomic rendering software for procedural planning (Figure 1).

**Figure 2:**
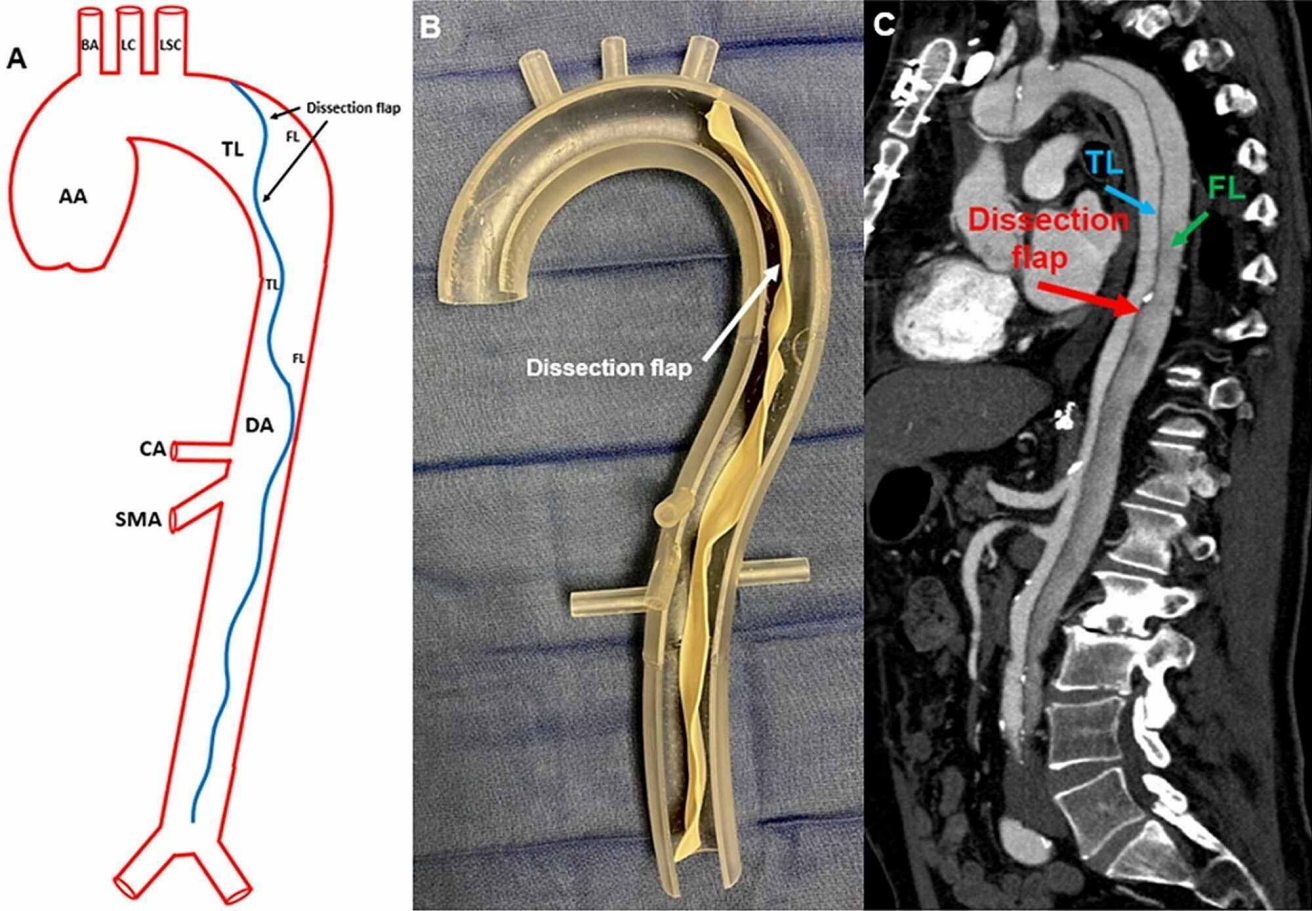
Stanford B Aortic Dissection An illustration of a Stanford B aortic dissection, originating immediately distal to the LSC to the aortic bifurcation, is shown (A). The AA, BA, LC, TL, and FL, dissection flap (blue line indicated by black arrows), DA, CA, and SMA are identified. A three-dimensional acrylic model of a Stanford B aortic dissection with an indwelling dissection flap (white arrow) is shown (B). The flap originates immediately distal to the left subclavian artery and terminates in the region of the aortic bifurcation. Computed tomography angiography of a Stanford B aortic dissection in the “candy cane,” or sagittal view is shown (C). The true lumen (blue arrow), false lumen (green arrow), and dissection flap (red arrow) are identified. The dissection extends immediately distal to the left subclavian artery, and to the aortic bifurcation. The true lumen supplies flow to the celiac axis and superior mesenteric artery. LSC: left subclavian artery; AA: ascending aorta; BA: brachiocephalic artery; LC: left carotid artery; TL: dissection true lumen; FL: false lumen; DA: descending aorta; CA: celiac axis; SMA: superior mesenteric artery

Stanford A aortic dissections are reserved for surgical intervention but may receive TEVAR post-operatively for the Stanford B aortic dissection portion. The basis for the covered and uncovered/open cell stent hybrid technique involves treatment of a completely dissected descending thoracic and abdominal aorta, originating immediately distal to the left subclavian artery, and terminating at the aortic bifurcation (Figure 2).

**Figure 3:**
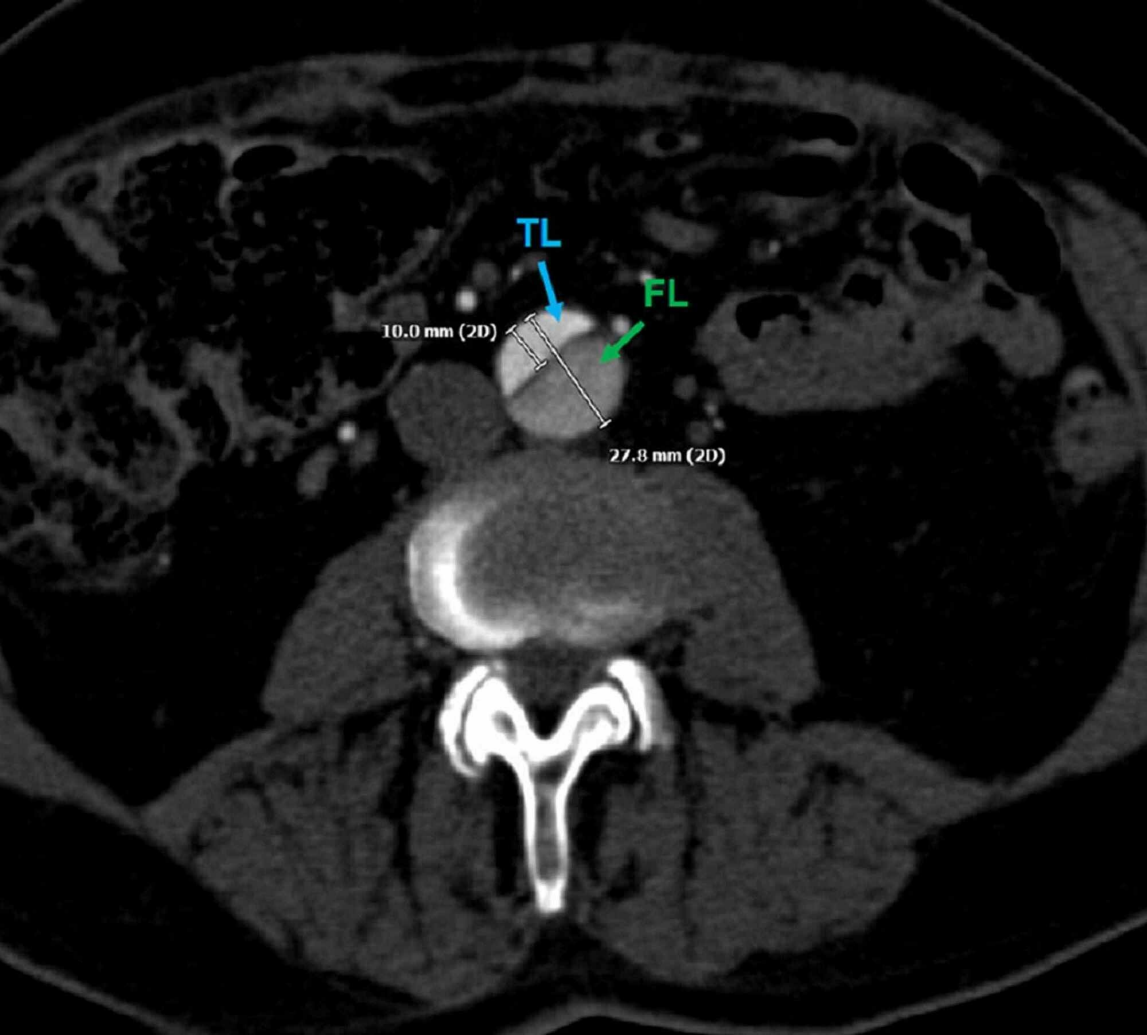
Axial Computed Tomography Angiography of a Pre-Procedural Stanford B Aortic Dissection A pre-procedural axial computed tomography angiogram shows the caliber sizes of the respective true (blue arrow) and false (green arrow) dissection lumens. The aortic diameter measures 27.8 millimeters in diameter at this level. The false lumen measures 17.8 millimeters and is noticeably larger than the true lumen, measuring 10 millimeters.

After a multispecialty consensus is made for treatment planning, the patient can be brought to the interventional radiology suite for TEVAR. A pre-procedural contrast axial CT angiography (CTA) of the abdomen identifies the patient’s dissected abdominal aorta with parallel true and false lumens. The expansion of the false lumen impedes the patency of the true lumen, resulting in a larger caliber false lumen (Figure 3).

**Figure 4:**
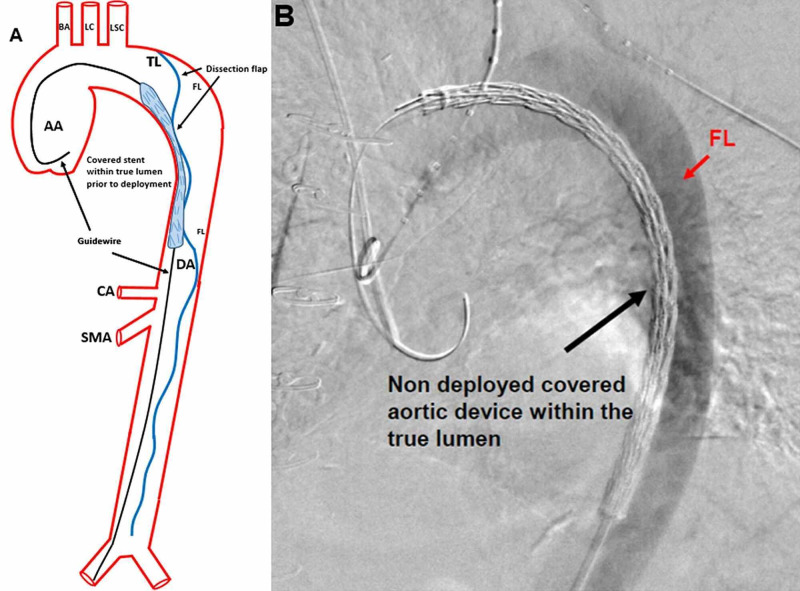
Thoracic Endovascular Aortic Repair of a Stanford B Aortic Dissection An illustration of the covered endograft being advanced over a stiff guidewire into the thoracic aorta before deployment is shown (A). A guidewire (black arrows) was positioned from the right femoral artery and was advanced to the AA. The covered aortic stent is within the true lumen. The BA, LC, LSC, TL, and FL, dissection flap (blue line indicated by black arrows), DA, CA, and SMA are identified. An intraoperative fluoroscopic guided angiogram shows the covered aortic device before deployment within the true lumen (black arrow), advanced over a stiff guidewire (B). Contrast can be identified within the false lumen (red arrow), abutting the non-deployed covered aortic device within the true lumen. AA: ascending aorta; BA: brachiocephalic artery; LC: left carotid artery; LSC: left subclavian artery; TL: dissection true lumen; FL: false lumen; DA: descending aorta; CA: celiac axis; SMA: superior mesenteric artery

In the interventional radiology suite, the patient’s left or right femoral artery is accessed using a micropuncture needle. A guidewire is introduced, and a sheath is positioned within the accessed femoral artery. A Lunderquist® (Cook Medical, Bloomington, IN) guidewire is then advanced into the femoral artery access, traversed through the abdominal aorta, and positioned at the aortic root with the assistance of intravascular ultrasound. This guidewire is used to support the covered endograft that will be advanced over it into the thoracic aorta (Figure 4).

**Figure 5:**
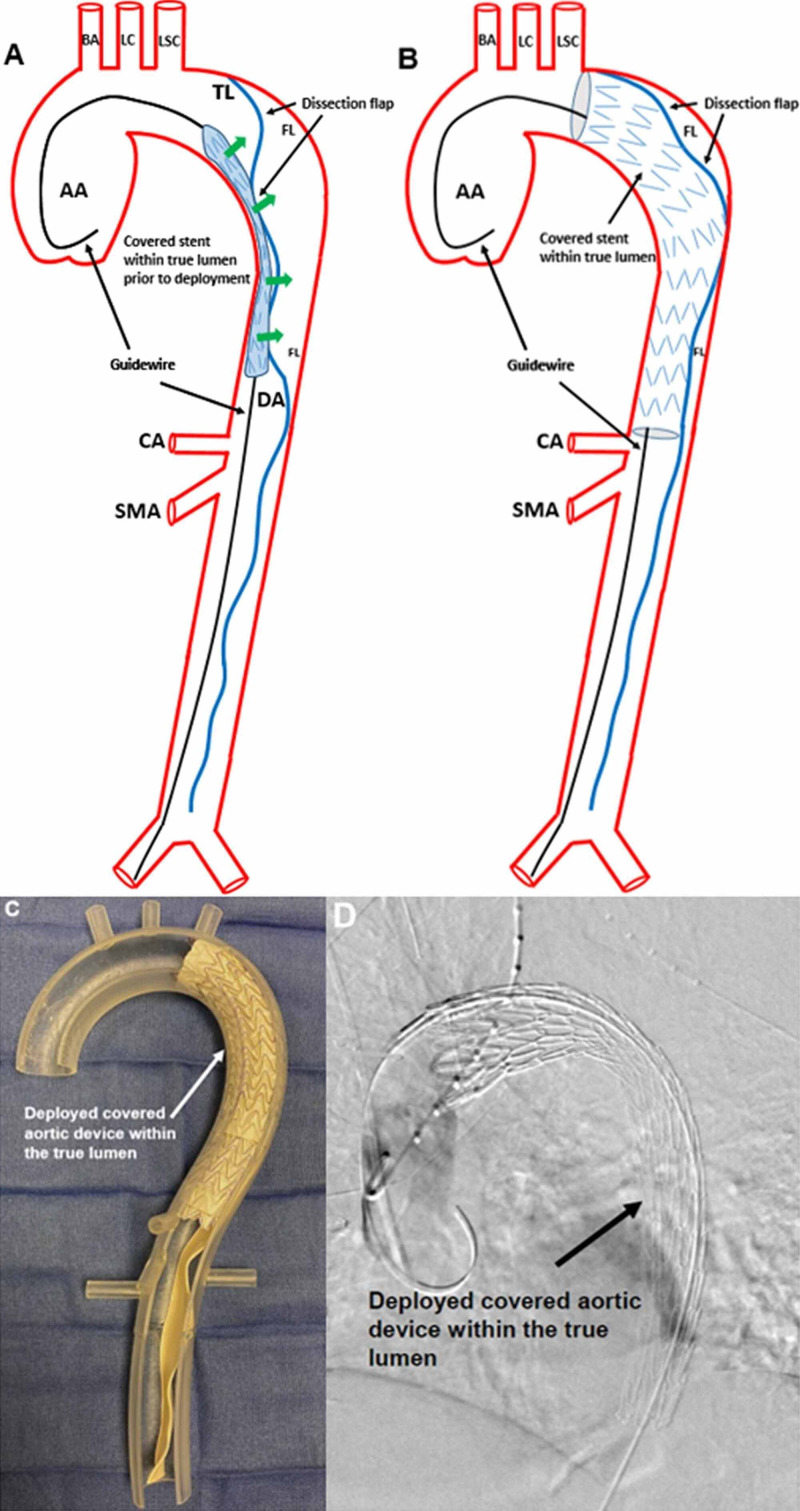
Radial Force of the Covered Endograft to Exclude the False Lumen An illustration of the covered endograft before deployment is shown (A). As the operator deploys the covered endograft, the radial force displaces the false lumen towards the aortic wall (green arrows). The guidewire (black arrows), AA, BA, LC, LSC, TL, and FL, dissection flap (blue line indicated by black arrows), DA, CA, and SMA are identified. An illustration following the deployment of the covered endograft shows the expansion of the covered endograft within the true lumen (B). The false lumen and dissection flap remain but will continue to diminish over time due to the continuous radial force applied by the covered endograft (black arrow). The distal landing zone of the covered endograft is just above the celiac axis. The guidewire (black arrows), AA, BA, LC, LSC, FL, dissection flap (blue line indicated by black arrows), CA, and SMA are identified. A three-dimensional acrylic model with a deployed covered aortic device within the true lumen is shown (C). The deployed covered aortic device (white arrow) excludes the proximal portion of the dissection flap, but the distal visceral dissection remains. An intraoperative fluoroscopic guided angiogram shows the deployed covered aortic device (black arrow) within the true lumen (D). AA: ascending aorta; BA: brachiocephalic artery; LC: left carotid artery; LSC: left subclavian artery; TL: dissection true lumen; FL: false lumen; DA: descending aorta; CA: celiac axis; SMA: superior mesenteric artery

A TAG® thoracic endoprosthesis device (W.L. Gore Incorporated, Newark, DE) is then advanced over the guidewire and deployed immediately distal to the left subclavian artery, where the aortic dissection originates. The constant radial force of the covered endograft displaces the false lumen towards the aortic wall, causing near-exclusion to the level of the celiac axis (Figure 5).

**Figure 6:**
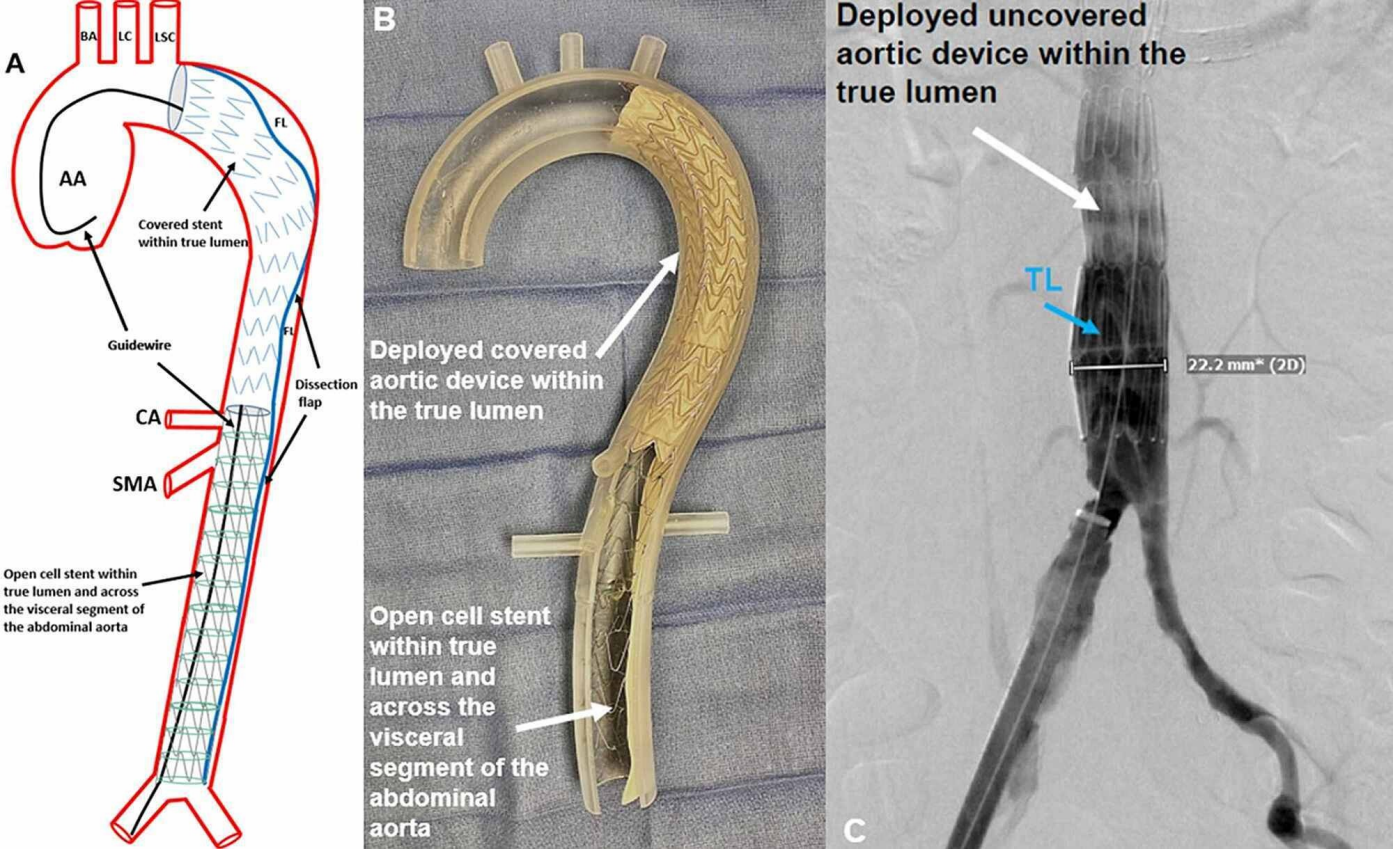
Bare Metal/Open Cell Exclusion of the False Lumen Dissection in the Visceral Segment and Abdominal Aorta An illustration of the bare metal/open cell stent deployed within the body of the covered endograft is shown (A). The expanded covered endograft within the true lumen is identified (black arrow). The bare metal/open cell stent is within the true lumen and traverses the visceral segment of the abdominal aorta (black arrow). Perfusion of the visceral segment is maintained as a result of an open cell configuration. A combination of the covered endograft and bare metal/open cell stent reduces the false lumen. The guidewire (black arrows), AA, BA, LC, LSC, dissection FL, dissection flap (blue line indicated by black arrows), CA, and SMA are identified. A three-dimensional acrylic model with both the deployed covered aortic device within the true lumen (white arrow), and bare metal/open cell stent within the true lumen (white arrow) is shown (B). An intraoperative fluoroscopic guided angiogram of the bare metal/open cell stent at the distal landing zone is shown (C). The deployed uncovered aortic device (white arrow) is within the true lumen (blue arrow), measuring 22.2 millimeters in diameter, and terminating just above the aortic bifurcation. AA: ascending aorta; BA: brachiocephalic artery; LC: left carotid artery; LSC: left subclavian artery; TL: dissection true lumen; FL: false lumen; DA: descending aorta; CA: celiac axis; SMA: superior mesenteric artery

A Zenith® (Cook Medical, Bloomington, IN) visceral aortic dissection open cell/bare metal stent is then advanced over the guidewire overlapping the covered endograft. The open cell/bare metal stent serves as the uncovered portion of the covered and uncovered stent hybrid technique. With consideration for positioning, the visceral aortic bare metal stent is deployed within the covered endograft at the level of the celiac axis. The distal end is positioned immediately above the aortic bifurcation (Figure 6). Flow to the visceral segments is preserved due to the passage of blood through the metal struts. The radial force of the covered endograft is evenly distributed throughout the entire descending thoracic and abdominal aorta. An immediate post-procedural angiogram shows the near-complete exclusion of the false lumen above the aortic bifurcation (Figure 6).

**Figure 7:**
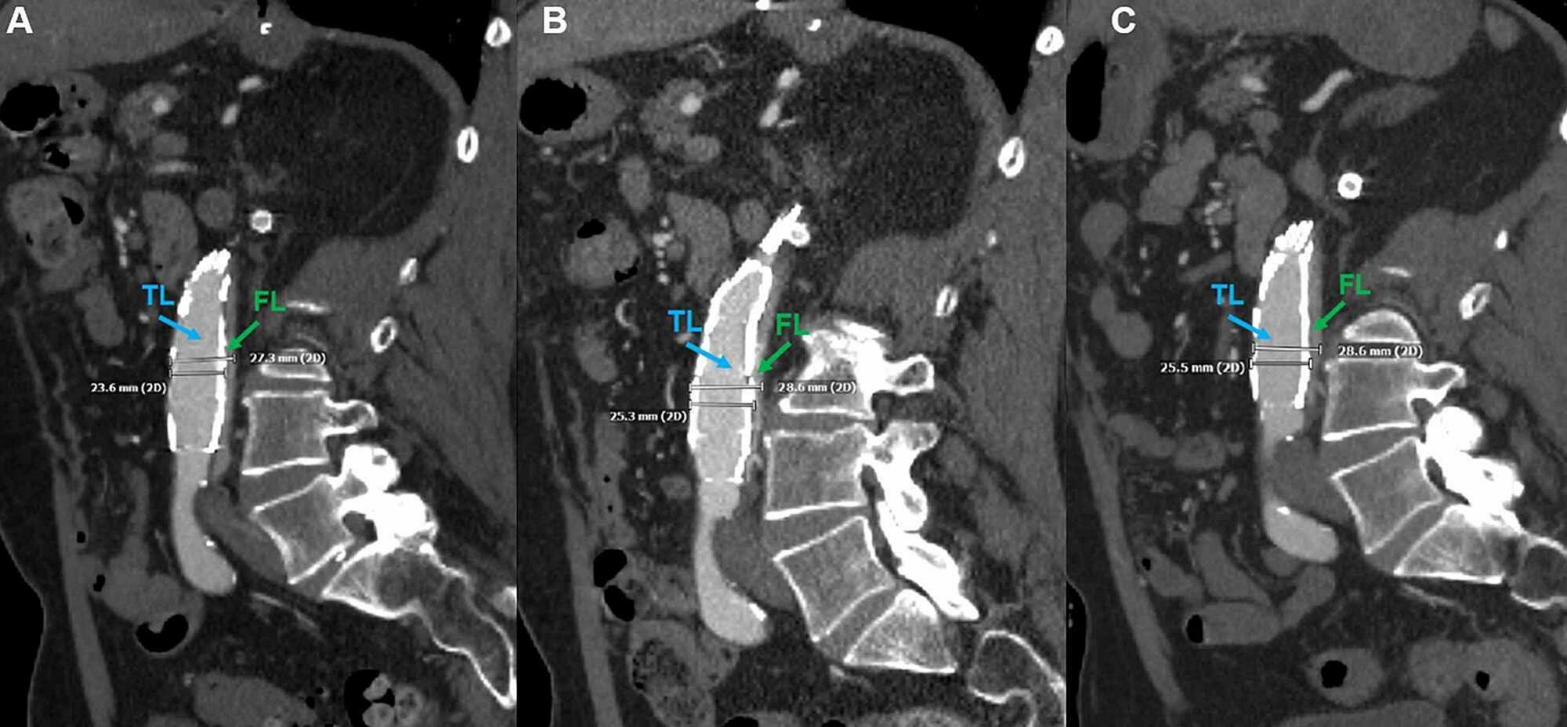
Post-Procedural Short and Mid-Term Surveillance A one month post-procedural sagittal computed tomography angiogram shows the bare metal/open cell stent within the true lumen (blue arrow), excluding the false lumen (green arrow), and terminating appropriately just above the aortic bifurcation (A). The true lumen is noticeably larger in diameter than the false lumen, measuring 23.6 millimeters. A four-month post-procedural sagittal computed tomography angiogram of the same patient shows the bare metal/open cell stent within the true lumen (blue arrow), continuing to exclude the false lumen (green arrow), and terminating appropriately just above the aortic bifurcation (B). The expansile capability of nitinol within the open cell/bare metal stent has increased the true lumen diameter to 25.3 millimeters, while further diminishing the size of the false lumen after four months. A nine-month post-procedural sagittal computed tomography angiogram of the same patient again shows the bare metal/open cell stent within the true lumen (blue arrow), continuing to exclude the false lumen (green arrow), and terminating appropriately just above the aortic bifurcation (C). There is a marginal increase in the true lumen diameter, measuring 25.5 millimeters in comparison to the four-month post-procedural computed tomography angiogram (Figure 7B).

One, four, and nine months of surveillance CTA demonstrate the continued expansion of the visceral aortic bare metal stent within the true lumen, identified by the increased true lumen caliber (Figure 7).

**Figure 8:**
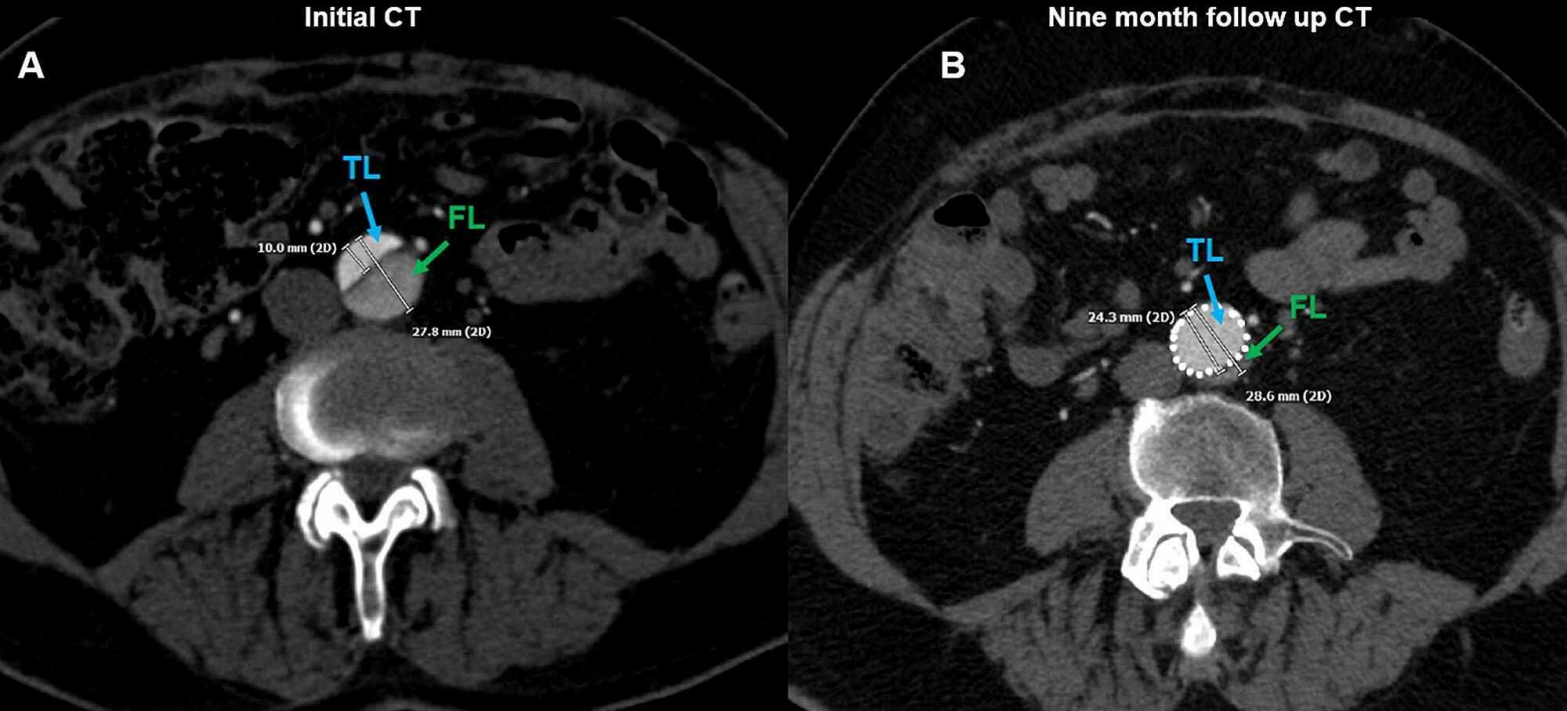
Mid-Term Surveillance True Luminal Patency of Thoracic Endovascular Aortic Repair The initial pre-procedural axial CT angiogram (Figure 3) shows the caliber sizes of the respective true (blue arrow) and false (green arrow) dissection lumens (A). The aortic diameter measures 27.8 millimeters in diameter at this level. As demonstrated (Figure 3) the false lumen measures 17.8 millimeters and is noticeably larger than the true lumen, measuring 10 millimeters. A comparison nine-month post-procedural axial CT angiogram of the same patient shows the caliber sizes of the respective true (blue arrow) and false (green arrow) dissection lumens after thoracic endovascular aortic repair with the covered and uncovered stent hybrid technique (B). The true lumen is noticeably larger in diameter than the false lumen, measuring 24.3 millimeters. The open cell/bare metal stent continues to exclude the false lumen after nine months.

The nine-month surveillance period shows the true lumen caliber remaining the same diameter or greater due to the persistence of circumferential radial force, which is evenly distributed throughout the thoracic and abdominal aorta. Additionally, the expansile memory of nitinol within the covered endograft and open stent allows for the expansion of the true lumen diameter. A comparison of the pre-procedural and nine-month mid-term surveillance contrast axial CTA of the abdomen shows true luminal patency at the level of the inferior mesenteric artery (Figure 8).

Both the covered endograft and open cell stent will continually expand outward, secondary to applied radial force onto the false lumen. Patients with nonocclusive visceral segments, such as within the celiac axis, renal arteries, and superior mesenteric artery, received angioplasty with Viabahn® endograft (W.L. Gore Incorporated, Newark, NE) before placement of the visceral aortic bare metal stent. Patients are monitored in the post-procedural surveillance period by a multispecialty team.

## Results

A retrospective analysis of nine patients underwent post-procedural surveillance for TEVAR of Stanford B aortic dissections, using the described covered and uncovered stent hybrid technique. A collaborative trauma center with access to multidisciplinary interventional radiology, cardiology, and cardiothoracic surgery team was integral in the documented interventional success, during the peri-procedural periods. The demographic of the patient group included various ethnicities and vasculopathic comorbidities (Table 1).

**Table 1:** Patient Demographics and Comorbidities The demographics and comorbidities of patients treated in this retrospective analysis with the covered and uncovered stent hybrid technique are shown.

Mean Age (years) (range)	49 (29-68)
Male	9 (100%)
Race	
White	5 (56%)
Black	2 (22%)
Hispanic	1 (11%)
Other	1 (11%)
Comorbidities	
Hypertension	9 (100%)
Diabetes	3 (33%)
Smoking	5 (56%)
Coronary Artery Disease	3 (33%)
Congestive Heart Failure	2 (22%)
Chronic Kidney Disease	3 (33%)
Marfan Syndrome	1 (11%)

**Figure 9:**
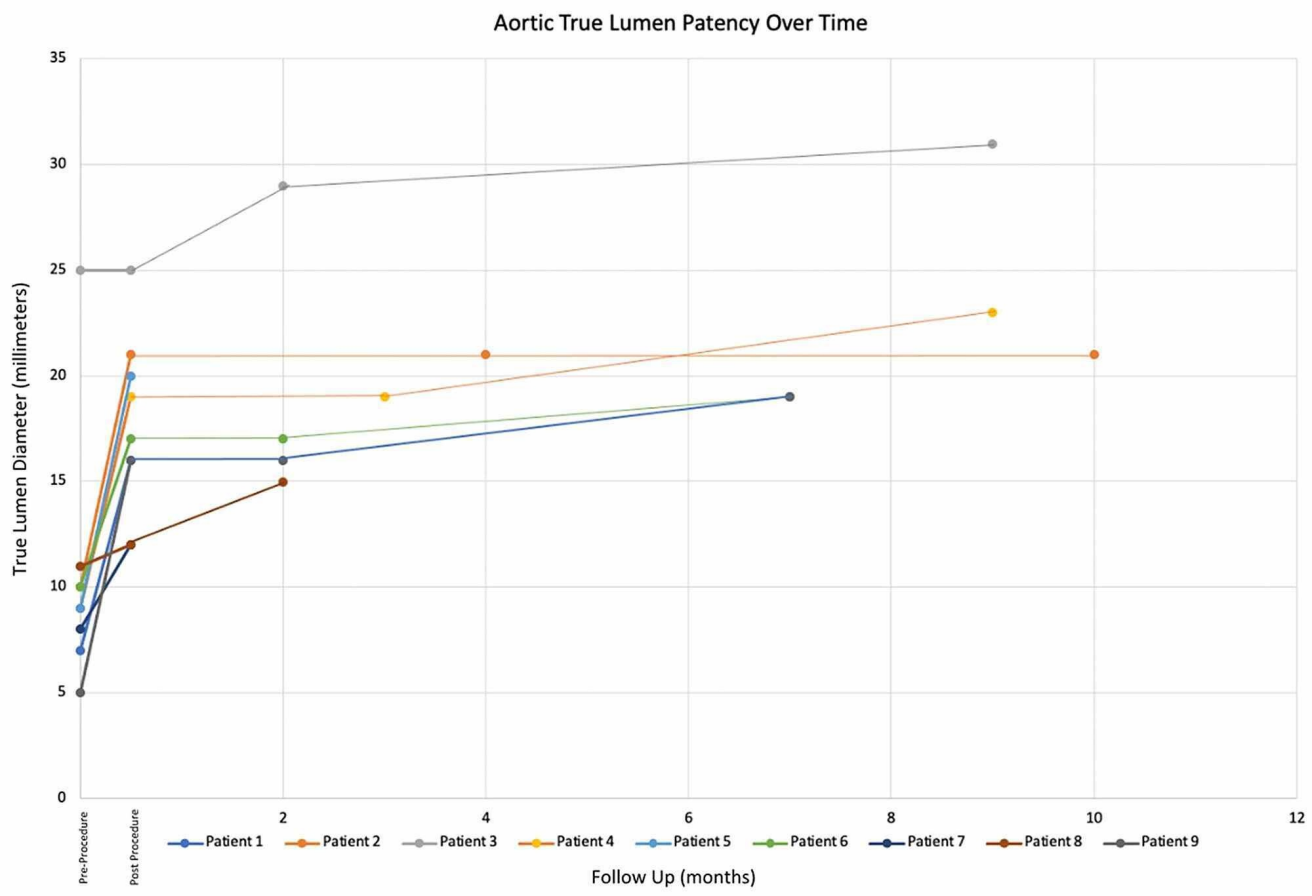
Aortic True Lumen Patency Over Time The graph shows true lumen patency throughout the surveillance period. All patients in the immediate post-procedural period demonstrated a noticeable increase in true lumen patency. For the patients able to follow-up in the short and mid-term surveillance periods, all patient aortic true lumens remained patent, five showing continued expansion.

100% of the patients had hypertension. In all nine patients, 100% of the immediate and mid-term patency rates were reported (Figure 9).

The surveillance period was dependent on access to patient follow-up and varied between one and four months in the short-term, and seven and 10 months in the mid-term (Table 2).

**Table 2:** Retrospective Analysis of Covered and Uncovered Aortic Stent Patency The retrospective data of the covered and uncovered stent hybrid technique in the immediate, short, and mid-term surveillance periods are shown. The extent of each patient’s Stanford B aortic dissection, pre-procedural symptoms, specific endovascular approach, the necessity for visceral stenting, pre-procedural true lumen patency at the level of the inferior mesenteric artery, immediate post-procedural true lumen diameter, short-term true lumen diameter, mid-term true lumen diameter, and survival are shown. With the exception of one patient, the pre-procedural dissection true lumen was measured at the level of the inferior mesenteric artery. Three patients were lost to follow-up during the severe acute respiratory syndrome-coronavirus-2 pandemic and do not have surveillance patency data. All patients had immediate post-procedural aortic true lumen patency and exclusion of the false lumen. Except for one patient, all patients survived in the mid-term post-procedural period. *Measurement of the aortic true lumen at the level of the celiac axis. TEVAR: thoracic endovascular aortic repair; SMA: superior mesenteric artery; IMA: inferior mesenteric artery

Type of Aortic Dissection	Pre-Procedural Aortic Dissection Induced Malperfusion	Procedure	Visceral Stenting	True Lumen at IMA (millimeters)	True Lumen Immediate Post-Procedure at IMA (millimeters)	Surveillance (millimeters)	Surveillance (millimeters)	Outcome
History of Stanford type A aortic dissection repair; new Stanford type B aortic dissection to iliac artery bifurcation	None	TEVAR; Cook^®^ open cell stent	None	7	16	None	None	Alive
Stanford type B aortic dissection to iliac artery bifurcation	None	TEVAR; Cook^®^ open cell stent	None	10	21	21 (4 months)	21 (10 months)	Alive
Stanford type B aortic dissection to SMA^+^	None	TEVAR, carotid-subclavian artery bypass, Cook^®^ open cell stent	Celiac axis	25^*^	25^*^	29^*^ (2 months)	31^*^ (9 months)	Alive
Stanford type A aortic dissection to iliac artery bifurcation	Right coronary artery	Ascending aorta replacement; second stage TEVAR, carotid-subclavian artery bypass, Cook^®^ open cell stent	Left renal artery	9	19	19 (3 months)	23 (9 months)	Alive
Stanford type A aortic dissection to iliac artery bifurcation	Cardiac tamponade	Ascending aorta replacement; second stage TEVAR, Cook^®^ open cell stent	Left renal artery	9	20	None	None	Alive
Stanford type B aortic dissection to iliac artery bifurcation	Left lower extremity ischemia	Right-to-left femoral-femoral artery bypass; second stage TEVAR, Cook^®^ open cell stent	Celiac axis, SMA, left renal artery	10	17	17 (2 months)	19 (7 months)	Death: cocaine overdose
Stanford type B aortic dissection to iliac artery bifurcation	None	TEVAR, Cook^®^ open cell stent	Celiac axis; SMA	8	12	None	None	Alive
Stanford type B aortic dissection to iliac artery bifurcation	Mesenteric ischemia	Exploratory laparotomy; second stage TEVAR, Cook^®^ open cell stent	Celiac axis; SMA	11	12	15 (1 month)	None	Alive
History of Stanford type A aortic dissection repair; new Stanford type B aortic dissection to iliac artery bifurcation	None	TEVAR, carotid-subclavian artery bypass, Cook^®^ open cell stent	Celiac axis; left renal artery	5	16	16 (1 month)	19 (7 months)	Alive

The widened range for short and mid-term surveillance resulted from patient safety considerations during the severe acute respiratory syndrome-coronavirus-2 pandemic and intermittent loss of patient follow-up. Aortic patency was generally measured with the difference between the control, in this case, the pre-procedural aortic true lumen diameter at the level of the inferior mesenteric artery, and in the post-procedural aortic true lumen diameter at the level of the inferior mesenteric artery. Patency in one patient was measured based on the pre- and post-procedural aortic true lumen diameter at the level of the celiac axis. The differences between pre- and post-procedural patency at varying visceral segments were based on the extent of the aortic dissection. Seventy-eight percent of the patient group required visceral segment intervention. The visceral intervention included endovascular stenting of the renal arteries, celiac axis, superior mesenteric artery, or a combination of these segments, in addition to TEVAR for Stanford B aortic dissection. Stenting was performed on patients’ visceral segments that shared perfusion from both the true and false aortic lumens. Post-stented vessels excluded the false lumen, limiting the visceral vascular supply to only the true aortic lumen. Forty-four percent of the patients had pre-existing repairs for Stanford A aortic dissection prior to TEVAR for Stanford B aortic dissection. There was only one complication reported in the surveillance period; one patient was reported to have a type 1A endoleak. This patient’s type 1A endoleak was revised and retained true luminal patency in the mid-term surveillance period. There was an 89% patient survival rate in the mid-term surveillance period; one patient died from an unrelated incident, separate from endovascular or medical management.

## Discussion

Medical management and lifestyle modifications have been the standard of treatment for Stanford B aortic dissections for decades [2]. It has been documented that the complication rate from untreated Stanford B aortic dissections without medical management is approximately 75% [18]. The most common complication of Stanford B aortic dissections without medical management is dissection expansion into the visceral segment, causing visceral organ malperfusion [18].

Despite the timely diagnosis and medical management, there is a 10-15% early mortality rate, which as previously discussed, contributes to a 30% overall mortality in patients with Stanford B aortic dissections [9]. Additionally, 50% of these same patients receiving appropriate medical management will develop aneurysmal degeneration, which has poor long-term survival rates [19]. Though the outcomes for Stanford B aortic dissections are poor due to the detriment of pre-existing comorbidities, regardless of symptomatology, TEVAR serves as a barrier from the poor prognostic alternative that is inherent in surgical management. Therefore, it would be pertinent to discuss the benefits that TEVAR serves, in addition to medical management.

The recent evidence of TEVAR for Stanford B aortic dissections demonstrates the promise for improved prognosis over medical management as the standard of care. Endovascular devices have been considered an ancillary alternative to treatment for any etiology of the aortic disease since the first TEVAR for aortic aneurysms were introduced in 1994, as an addition to conventional medical management [20]. At that time, the immediate limitations for endovascular repair were evident, and have been addressed and refined in the last two decades. Since then, TEVAR has become comparable and even superior to the standard of care, as device technology has evolved. The rapid expansion for both improved quality of devices and the quantity of devices available allows for the improved success rate of endovascular intervention. The favorable outcomes in the covered and uncovered stent hybrid technique described directly results in the progressive refinement of device quality and availability.

Device failure can be identified as a prominent cause for treatment inadequacy when treating Stanford B aortic dissections in the past. Among the first documented TEVAR attempted, one called for the use of an off label “home made,” covered endovascular device with an interwoven dacron cuff, in addition to open thoracic aortic repair [21]. It is reported that the 30-day mortality rate for these patients in this study was 9%, mostly due to cerebrovascular accident and myocardial infarction.

The advent of other endovascular devices since then has improved upon targeted treatment approaches for Stanford B aortic dissections above the visceral segment. This yields progressively positive prognostic results in long-term surveillance. Devices can be the cause of post-intervention complications, such as aneurysm formation, failure of dissection thrombosis, expansion of the dissection lumen, and endoleak formation [22]. In order to optimize outcomes for patients with Stanford B aortic dissections, the indications for TEVAR should be clearly delineated. Accurate stratification will guide the interventionist in choosing both the appropriate treatment method and device that will remain patent in post-treatment surveillance. Foundationally, patients should receive TEVAR in addition to medical management if they decompensate or become medically unstable within two weeks of diagnosis [23]. Severe acute dissection related abdominal pain is also pertinent, and should not be ignored within the first two weeks of diagnosis. Such pain should be followed-up with an urgent abdominal ultrasound examination to monitor for dissection expansion [23]. Other signs and symptoms that lead to hemodynamic instability in a patient recently diagnosed with a Stanford B aortic dissection, may include aortic aneurysmal expansion, dissection expansion, and visceral organ malperfusion [24]. The most encompassing complication that should be addressed with TEVAR in patients with Stanford B aortic dissections, is visceral organ malperfusion, as a result of false lumen dissection expansion [24]. Combined, these acute signs and symptoms account for the acuity of approximately 25% of Stanford B aortic dissections, which leads to much higher mortality than chronic Stanford B aortic dissections [23]. Therefore, it is equally pertinent for the competency of the interventionist to match the quality of the chosen device. 

Currently, there are four FDA-approved endovascular grafts for TEVAR. These four grafts are the Zenith® Alpha (Cook Medical, Bloomington, IN) thoracic endovascular graft, the TAG® thoracic endoprosthesis device, the RelayPlus® (Bolton Medical Incorporated, Sunrise, FL) endovascular graft system, and the Valiant® (Medtronic, Minneapolis, Minnesota) thoracic stent graft. All these endovascular grafts are covered and are designed to be deployed proximal to the visceral segment. TEVAR for patients in our retrospective analysis received the TAG® thoracic endoprosthesis device. This specific covered endograft was shown to be superior in 30-day mortality, shortened hospital length of stay, decreased cerebrovascular accidents, and major adverse clinical events, compared to open surgical repair of the thoracic aorta, for dissections distal to the origin of the left subclavian artery [25]. A single nonrandomized study at ten sites demonstrated technical success in 100% of the patients with low rates of aortic related mortality within 30 days [26]. A combination of the TAG® thoracic endoprosthesis device and Zenith® visceral aortic bare metal stent, yields additive prognostic value of TEVAR for graft patency in the visceral segment. Though certain covered endovascular grafts have proven various levels of superiority when compared with surgical intervention, none are without their caveats. For example, fenestrated endograft systems can be utilized to accommodate all variations of patient anatomy, but the complication rate in the peri-procedural setting and revision rate is high [27]. In general, the FDA-approved endografts for TEVAR show promising outcomes in maintaining aortic true lumen patency, when used to repair Stanford B aortic dissections. However, these devices have limitations with varying risks in the peri-procedural setting. Interventionists should be well acquainted with these device limitations before proceeding with endovascular management.

Adequate angiographic outcomes are often improved, but not optimally achieved within the visceral segment, even after the utilization of a covered and uncovered stent hybrid technique, as previously described. This can explain why 78% of our retrospectively analyzed patient group required the deployment of visceral endografts. As a result of a combination of the covered and uncovered stent hybrid technique and visceral endograft repair, angiographic results were optimal in the patient group and continued to be in the mid-term surveillance period. The technical success we demonstrated may be the result of contributed attention to dissection characteristics that have often been neglected in the past. 

The pitfalls observed as TEVAR has been refined include the degree of anatomical tortuosity in the proximal aorta, endograft distribution of axial stress from the proximal landing zone, the distribution of radial force throughout the true lumen, the straightening of the aortic true lumen immediately proximal to the visceral abdominal aorta, the tapering endograft radial force in the distal landing zone, and the positioning of the endograft at the distal landing zone. The covered and uncovered stent hybrid technique we describe addresses all of these historic obstacles in TEVAR for Stanford B aortic dissections. Most importantly, the TEVAR we describe considers the positioning at the distal landing zone, which is crucial for aortic patency and perfusion within the visceral segment.

The current literature demonstrates that TEVAR for Stanford B aortic dissections has historically resulted in poor outcomes at the distal landing zone, as the graft tapers in the true lumen. The addition of a properly positioned overlapping distal open cell/bare metal stent, adds radial force for increased aortic true lumen size and patency. An uncovered bare metal stent when properly overlapped within the proximal covered endovascular graft assists in redistributing the axial force onto the proximal abdominal aorta and allowing for aortic true luminal patency. Additionally, the bare metal stent limited intimal dissection propagation into the visceral segment, which would otherwise lead to end-organ malperfusion. This specific complication has historically demonstrated the highest patient mortality rates among all associated with TEVAR [28,29]. Type 1 endoleaks are related to complications arising from the positioning of the proximal and distal landing zones [15,16]. These specific endograft complications are the result of blood flow around or outside of the graft lumen, which may result in the collapse of the aortic true lumen, thrombosis, de novo aortic dissection, or aortic aneurysm at the weakened luminal wall.

Type 1 endoleaks are failures of a positioned endograft occurring at the proximal segment (1A) and distal segment (1B). It is particularly important that these types of endoleaks are identified in the patient follow-up period because they are dependent on the procedural success and prognostic outcome [15,16]. Poor prognostic outcomes are more heavily weighted with type 1B endoleaks over type 1A endoleaks after TEVAR. The incidence and mortality of type 1A and 1B endoleaks in one study after TEVAR were 3.4% and 26.1%, respectively [30]. In that cohort, approximately 35% of the aortic endografts placed, led to type 1B endoleaks and distal aortic dissection. These particular patients in this study had a 25% mortality rate [30]. Similar to the malpositioning of the aortic endovascular graft at the distal landing zone, type 1B endoleaks occurring within the aortic endograft have much higher mortality than type 1A endoleaks, due to the high probability of aortic dissection expansion into the visceral segment. As mentioned, aortic dissection expansion into the visceral segment has historically demonstrated the highest patient mortality rates, where open surgical management has been the only option for repair with poor outcomes. Notably, the only complication observed from our retrospective data was a type 1A endoleak in the covered aortic endograft of one patient. However, a persistently favorable prognosis for this patient was demonstrated with aortic true luminal patency in the mid-term surveillance period. This may be attributed to the lower risk for complication compared to the high-risk mortality associated with type 1B aortic endograft endoleaks [30]. We can partially contribute to the success of the covered and uncovered stent hybrid technique, by securing the distal landing zone of the proximal covered endograft, and reducing the risk for aortic endograft type 1B endoleaks. We believe this technique prevented the propagation of an expanding distal aortic dissection into the visceral segment.

## Conclusions

Opposed to the current standard of care, the advent of TEVAR has resulted in improved treatment outcomes for Stanford B aortic dissections. However, despite the documented interventional success, the high complication and mortality rates inherent to Stanford B aortic dissections are additive with the peri-procedural complications associated with TEVAR. We describe a covered and uncovered stent hybrid technique that considers the historically neglected aspects of TEVAR, resulting in a 100% technical success rate.
